# Supply chain carbon reduction considering consumer skepticism and blockchain technology under the cap-and-trade policy

**DOI:** 10.1371/journal.pone.0345379

**Published:** 2026-04-17

**Authors:** Peng-peng Yuan, Qingsong Wang

**Affiliations:** 1 School of Digital Economics and Management, Wuxi University, Wuxi, China; 2 School of Digital Economics and Management, Suzhou City University, Suzhou, China; East China Normal University, CHINA

## Abstract

In the context of low-carbon development, both firms and consumers can contribute to environmental protection. Given that consumers are often skeptical of corporate claims regarding product carbon reductions, this study explores how manufacturers can leverage blockchain technology to invest in carbon abatement under carbon cap-and-trade policies. Then, this study develops three game models within a supply chain involving a manufacturer and a socially responsible retailer to explore firms’ abatement and operational strategies. The model design is based on existing literature as well as real-world corporate practices, cap-and-trade policies, and blockchain transparency mechanisms. The study finds that unit abatement level is negatively correlated with consumer skepticism and positively correlated with the retailer’s corporate social responsibility. When the unit application cost of blockchain is below a certain threshold, manufacturers should adopt blockchain technology to mitigate consumer skepticism, thereby increasing product abatement levels and improving profits. Moreover, this threshold increases with market size and the degree of consumer skepticism. Retailers’ socially responsible behavior can enhance both the manufacturer’s unit abatement level and profitability, while stronger consumer preferences for low-carbon products or higher carbon prices can further motivate retailers to assume social responsibility. When the cost-sharing ratio is at a low level, both firms can achieve a win–win outcome in terms of profitability and environmental performance. The optimal cost-sharing ratio is positively related to the level of consumer low-carbon preference and the level of social responsibility. These findings provide theoretical insights and practical guidance for promoting credible carbon reduction strategies in supply chains.

## 1. Introduction

In recent years, excessive greenhouse gas emissions, particularly carbon dioxide, have intensified climate challenges [1]. To mitigate these impacts, policymakers have implemented various regulatory instruments, such as carbon taxes, green subsidies, and cap-and-trade schemes [2–5]. Among them, the carbon cap-and-trade policy (CTP) is widely recognized as one of the most effective mechanisms [6]. Under CTP, the government allocates emission allowances (“caps”) to firms; those exceeding their quotas must purchase additional allowances in the carbon market, while firms with surpluses may sell them to generate revenue [7]. Driven by carbon policies, many firms now integrate carbon considerations into supply chain decisions. Such practices not only help firms meet stakeholders’ environmental expectations but also demonstrate corporate social responsibility, particularly in addressing concerns about product carbon footprints [8–10]. However, information asymmetry and the prevalence of “greenwashing” have fostered consumer skepticism regarding actual product emissions, exemplified by the 2015 Volkswagen scandal [11–13]. This skepticism may weaken consumer preference for low-carbon products, thereby influencing firms’ operations and abatement strategies.

Blockchain technology, with its decentralization, tamper-resistance, and traceability, enables consumers to track product carbon footprints and is increasingly adopted by firms to enhance transparency and mitigate consumer skepticism [14–16]. For instance, the State Grid Corporation of China leverages blockchain to address issues such as limited traceability, regulatory difficulties, and low credibility in green electricity trading. However, the adoption of blockchain involves additional costs and may crowd out part of the resources allocated to carbon abatement, thereby affecting firms’ compliance with the CTP [17]. Moreover, the benefits of blockchain and abatement are often shared across the supply chain. These considerations raise several critical questions: under what conditions can blockchain adoption simultaneously mitigate consumer skepticism, improve abatement performance, and enhance corporate profitability? How do CTPs, consumer skepticism, and retailers’ social responsibility shape firms’ operational and abatement decisions? To what extent are retailers willing to share the costs of blockchain adoption and carbon abatement, and what cost-sharing strategies are most effective?

Building on the preceding discussion, this study examines a supply chain comprising a carbon-abating manufacturer and a socially responsible retailer. The manufacturer is responsible for production and carbon abatement, and adopts blockchain technology to enhance the credibility and transparency of carbon reduction information, thereby mitigating consumer skepticism. The retailer demonstrates social responsibility by valuing consumer surplus and is incentivized to share the costs of the manufacturer’s carbon abatement and blockchain adoption [18]. Within this framework, three game-theoretic models are developed to address the above research questions: (1) the baseline model (N model), in which the manufacturer does not adopt blockchain and the retailer does not share costs; (2) the blockchain model (B model), in which the manufacturer adopts blockchain but the retailer does not share costs; and (3) the cost-sharing model (BS model), in which the retailer shares to the manufacturer’s carbon abatement and blockchain costs. For practical considerations, the BS model is further extended into two scenarios: one in which the retailer determines the cost-sharing ratio, and another in which it is negotiated between the two parties. The models are analyzed through parameter analysis, comparative evaluation, and numerical simulations, ultimately yielding key insights and managerial implications.

The innovation of this study is mainly reflected in three aspects. First, while many existing studies assume that carbon abatement directly generates product premiums or market expansion, they often neglect the role of consumer skepticism toward firms’ carbon reduction claims. This study explicitly incorporates consumer skepticism and examines its impact on firms’ decision-making. Second, to address consumer skepticism, the study investigates the role of blockchain in recording and verifying carbon reduction information, and identifies the adoption threshold of blockchain from the perspectives of abatement levels and manufacturer profitability. Third, in the context of CTPs, the social responsibility behavior of retailers and their cost-sharing decisions have been underexplored. This research integrates retailer social responsibility into the supply chain framework to examine its influence on operational and abatement decisions, and proposes a novel cost-sharing contract aimed at mitigating consumer skepticism, enhancing unit abatement level, and improving the profitability of supply chain members.

The remainder of this study is organized as follows. Section 2 reviews the relevant literature. Section 3 introduces the model assumptions and defines the key symbols. Section 4 presents the construction, solution, and analysis of the decision models. Section 5 provides a series of numerical analysis of the relevant findings. Finally, Section 6 summarizes the conclusions and discusses managerial implications.

## 2. Literature review

The literature relevant to this study mainly includes three aspects: supply chain management under carbon policies (including corporate abatement and operations management), blockchain applications in supply chain carbon reduction (including improving transparency and reducing consumer skepticism), and the role of cost-sharing contracts in supply chains.

### 2.1. Supply chain management under carbon policies

Amid growing environmental urgency, research on supply chain carbon abatement and operations has developed rapidly, with particular attention to the role of climate policies [19,20]. Among various low-carbon policies—including CTP, carbon taxes, and subsidies—CTP is most closely related to the focus of this study. Within this domain, a substantial body of literature has examined the abatement and operational decisions of manufacturing firms. For example, Xu et al. [21] developed a supply chain consisting of a manufacturer that produces competing products and a retailer, derived the optimal total emissions and output for two products, and further analyzed the effects of carbon prices on firms’ optimal profits. Zhang et al. [22] examined how manufacturers’ low-carbon strategies change with carbon caps under three supply chain power structures (manufacturer-led, retailer-led, and vertical Nash). Similarly, Mondal and Giri [6] compared firms’ optimal strategies under centralized, decentralized, retailer-led revenue-sharing, and bargaining-based revenue-sharing structures within the CTP framework. Meanwhile, the competitive and cooperative dimensions of carbon abatement among firms have also garnered considerable scholarly attention. For instance, Liu et al. [19] explored the impact of manufacturers’ abatement investments on both their own profits and those of their competitors, concluding that such investments increase profits for both parties. Yang et al. [23] examined pricing and abatement strategies in two competing supply chains under the CTP and found that vertical cooperation enhances abatement levels while reducing retail prices. Sun et al. [24] analyzed manufacturers’ abatement choices under three modes—independent abatement, upstream supplier abatement, and abatement via energy service companies—regulated by the CTP.

In addition, several studies investigate how various factors within the low-carbon policy framework influence firms’ abatement, production, pricing, and profitability. For example, Wang et al. [25] examined the effects of government subsidies and retailers’ social responsibility actions on manufacturers’ abatement decisions under the CTP. They found that both factors contribute to higher abatement levels and increased manufacturer profits. Yuan et al. [26] explored encroachment and abatement under scenarios with and without retailers’ low-carbon investments, and found that when unit encroachment costs fall below a certain threshold, manufacturers tend to increase per-unit abatement levels following encroachment. Similarly, Li et al. [27] investigated the impact of cost-sharing mechanisms on manufacturers’ abatement efficiency and retailers’ willingness to share demand information. Their results suggested that when manufacturers exhibit high abatement efficiency, they can obtain demand information from retailers at no cost. Liu et al. [28] studied the impact of demand uncertainty on supply chain pricing decisions under the conditions of without CTP regulation, a grandfathering mechanism, and a benchmarking mechanism.

Most existing studies assume that consumers fully trust firms’ reported abatement levels and largely overlook consumer skepticism as well as the potential role of blockchain technology (e.g., [18–26]). In reality, issues such as corporate “greenwashing” and information asymmetry between buyers and sellers have raised widespread concerns about the credibility of firms’ abatement data. Such concerns may, in turn, influence consumers’ purchasing decisions as well as firms’ abatement and operational strategies. Distinct from prior research, this study explicitly incorporates consumer skepticism into the supply chain context and examines its impact on firms’ carbon abatement and operational strategies under the CTP. Furthermore, this study investigates the potential of blockchain technology as a mechanism to mitigate consumer skepticism in low-carbon supply chains.

### 2.2. Blockchain applications in supply chain carbon reduction

As a decentralized and distributed technology, blockchain can safeguard the privacy and integrity of information across all transaction stages [29,30]. Accordingly, many scholars have examined its potential to address trust issues in supply chains [31]. For example, Choi [32] examined the value of a traditional network operating model versus a blockchain-supported platform operating model for diamond authentication in the luxury supply chain. They found that reducing the cost of blockchain-based diamond authentication is beneficial to all supply chain members. Shen et al. [33] explored how permissioned blockchain can combat the copycat in the supply chain. Zhou et al. [34] investigated a supply chain composed of traditional and sustainable product manufacturers and demonstrated that blockchain adoption alleviates consumer skepticism regarding sustainability information. Their results indicated that blockchain-based information disclosure enhances consumer trust and improves retailer performance. Duan et al. [35] found that the improved transparency of the supply chain supported by blockchain technology can enhance consumers’ trust in the focal company and its stakeholders.

Beyond trust, scholars have also explored how blockchain adoption influences supply chain operations and member profitability. Yi et al. [36] showed that blockchain adoption in the new energy vehicle battery recycling supply chain improves recycling rates and raises the marginal profits of both manufacturers and retailers. Gupta et al. [37] examined the role of blockchain in enhancing the financial resilience of supply chains. Their study concluded that blockchain can improve the financial resilience of supply chains, particularly under environmental regulatory pressures. Given the non-negligible costs of blockchain adoption, another research stream investigates investment strategies. Fang et al. [38] explored the blockchain investment decisions of manufacturers and retailers in a platform supply chain and found that when the commission rate is at a moderate level, both parties expect to benefit from the other’s investment, ultimately resulting in losses for both sides due to the absence of blockchain investment. Liu et al. [39] examined the collaboration between core enterprises in adopting blockchain. The authors found that a hybrid contract, combining cost-sharing and revenue-sharing mechanisms, can incentivize firms to adopt blockchain and achieve coordination within the supply chain system.

In the domain of low-carbon supply chains, blockchain has been linked to sustainability outcomes. Xu et al. [40] investigated manufacturers’ green investment and coordination decisions in a supply chain with one manufacturer and one retailer, and highlighted that blockchain positively influences product greenness and increases the profits of manufacturers and platform operators. Li et al. [41] employed a mean-variance model to investigate the impact of demand information sharing on the carbon reduction effects of blockchain technology. The authors found that both information sharing and blockchain adoption enhance supply chain sustainability and improve member performance. Kang et al. [42] employed differential game theory to study the blockchain adoption and green production investment strategies of firms. The study showed that increased product information transparency can improve the digital operation level of the supply chain, carbon emission reduction, and overall profit. Ran and Duan [43] explored the impact of carbon emission misreporting and blockchain adoption on the abatement and operational decisions of competing manufacturers. Their study indicated that although blockchain adoption enhances supply chain profitability, it does not necessarily reduce carbon emissions. Jiang et al. [44] constructed Stackelberg game models with and without blockchain under manufacturer-led and retailer-led scenarios, and found that blockchain enables firms to maintain optimal profits only when carbon emission costs are relatively low.

Although blockchain has received increasing attention for information verification and product traceability, its role in carbon abatement remains underexplored. Existing studies have given limited consideration to its application in low-carbon supply chains, particularly regarding how blockchain can mitigate consumer skepticism toward firms’ abatement information and influence abatement strategies under the CTP (e.g., [33–39]). This study aims to enrich this research area by introducing blockchain as a mechanism to enhance the credibility of abatement information and examining its strategic effects on supply chain decisions, including carbon abatement levels, pricing, cost-sharing, and profitability. Meanwhile, it provides a novel perspective for blockchain-enabled low-carbon supply chain management.

### 2.3. The role of cost-sharing contracts in supply chains

Contracts have long been regarded as an effective mechanism to coordinate supply chains by reducing costs and improving operational efficiency. Among various contractual forms, cost-sharing contracts, revenue-sharing contracts, quantity discount contracts, and two-part tariff contracts are the most frequently studied. For example, Ji et al. [45] explored the production decisions of firms and the carbon cap strategies of the government in a two-stage supply chain based on a wholesale price contract and a revenue-sharing contract, respectively. In terms of cost sharing, Ghosh and Shah [46] explained the impact of cost-sharing contracts on supply chain members’ green initiative decisions. Xia et al. [47] analyzed the promotion and carbon reduction decisions of supply chain members with and without a cost-sharing contract, finding that the manufacturer’s cost-sharing behavior can enhance the retailer’s promotional efforts. He et al. [48] compared three strategies for service integrators to share supplier costs—no cost-sharing, abatement cost-sharing, and service cost-sharing—and found that the latter two improve benefits for all supply chain members. Beyond the contract forms themselves, scholars have also considered additional behavioral and contextual factors. Wang et al. [13] analyzed the impact of a cost-sharing contract with altruistic preferences on the profits and decisions of supply chain members. The study showed that this contract can increase the profitability and efficiency of small and medium-sized manufacturers. In the domain of low-carbon supply chains, Song et al. [49] analyzed how manufacturer cost-sharing affects retailers’ advertising, product greenness, and supply chain profits when fairness concerns are present. Their results indicated that manufacturers can enhance joint profitability and improve overall supply chain greenness through cost-sharing contracts. He et al. [50] employed a differential game model to study the dynamic abatement problem jointly undertaken by suppliers and manufacturers under both centralized and decentralized settings. They further designed a bilateral participation contract to achieve coordination within the supply chain. Taleizadeh et al. [51] examined how cost-sharing contracts influence new technology investment, carbon emission reduction, product sustainability, and quality enhancement in a dual-channel supply chain with one manufacturer and one retailer.

The aforementioned literature demonstrates that scholars have made substantial progress in the field of cost-sharing contracts (e.g., [45–49]). However, few existing studies comprehensively examine how cost-sharing mechanisms interact with blockchain adoption and consumer skepticism, thereby influencing firms’ carbon reduction and operational strategies under the CTP. In contrast, this study examines the impact of retailers’ cost-sharing on supply chain carbon abatement, blockchain adoption, and firm profitability. Moreover, it explores the differences in the optimal cost-sharing ratio under two different schemes: one in which the retailer determines the ratio, and the other in which the two parties negotiate the ratio through bargaining.

## 3. Methodology

### 3.1. Model descriptions

Under the CTP, this study describes a supply chain consisting of a manufacturer investing in carbon reduction and a socially responsible retailer. The wholesale price and retail price of the product are w and p respectively. However, given the presence of “greenwashing” or emission falsification in the market [12], this study assumes that consumers skeptical of the abatement levels disclosed by manufacturers. Parameter η denotes the level of consumer skepticism. To address consumer skepticism, the manufacturer adopts blockchain to record the abatement level of unit product [40]. Consumers can trace the abatement levels of each production link through the blockchain platform [16].

### 3.2. Decision sequence

**Fig 1:**
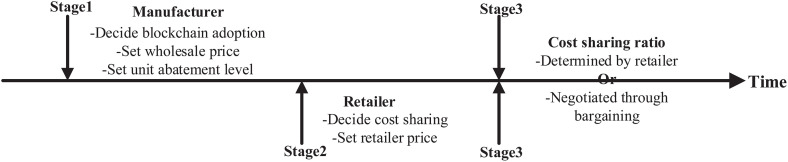
Decision-making sequence.

Fig 1 depicts the decision-making sequence of the two firms. In the first stage, the manufacturer decides whether to adopt blockchain and determines the wholesale price and unit abatement level. In the second stage, the retailer determines whether to engage in cost sharing and sets the retail price. In the final stage, the cost-sharing ratio is determined either unilaterally by the retailer or jointly through bargaining.

### 3.3. Fundamental assumption

Similar to Xia and Niu [52], this study assumes that the manufacturer’s unconstrained emission quota is E, and the initial emission per unit of product is e. The carbon credit trading price $p_{e}$ is determined by the carbon credit trading market [26]. The one-time investment for manufacturers to reduce emissions is $hx^{\text{2}}/2$, and x is the abatement level per unit of product (0<x<e) [53].

Referring to Panda [18], this study uses consumer surplus to characterize retailers’ social responsibility. The consumer surplus can be expressed as $CS=\int_{pmin}^{pmax}qdp=q^{\text{2}}/2$. Moreover, 0≤t≤1 indicates the retailer’s concern about consumer surplus, i.e., the retailer’s social responsibility level. A larger t indicates a higher level of social responsibility. t=0 indicates that the retailer is a pure profit maximizer; t=1 means that the retailer is a perfect socially responsible maximizer.

On the basis of Hong and Guo [54], the utility that consumers can obtain by consuming a unit of product is u=v−p+(1−η)bx. v represents the basic utility obtained by the consumer from a unit of the product, obeys a uniform distribution on [0,a]; $\eta_{\text{1}}=1-\eta$ is the degree of consumer trust in the abatement level; 0<b<1 is the low-carbon preference level of consumers [26]. Based on the above utility function and following the approach of Ryan et al. [55], the demand function can be derived as: $q=a-p+\eta_{\text{1}}bx$.

This study normalizes the production cost to 0 [52]. Furthermore, the blockchain application cost per unit product is denoted as c. To ensure that the profit functions are concave with respect to the decision variables, this study further assumes that $2h(1-t)(2-t)>(b+p_{e}{)}^{\text{2}}$ and λ<(1−t)/(3−2t). Similar assumptions are widely adopted in the field of operations management (e.g., [6,24]).

Consider the case where the social responsibility behavior is performed by the retailer. This study refers to Panda [18] and assumes that the retailer acts with the objective of maximizing the utility $U_{r}=\pi_{r}+tCS$, where $\pi_{r}$ is the retailer’s profit.

The subscripts m and r refer to the manufacturer and retailer. The superscripts N, B, and BS refer to the three modes, respectively. The notations and definitions of the main variables and parameters used in this study are summarized in Table 1.

**Table 1:** Main parameters and variables.

Notations	Definitions
Parameters	
h	Cost coefficient of abatement
E	Cap allocated to the manufacturer
η	Level of consumer skepticism, 0≤η≤1
a	Potential market size
b	Consumers’ low-carbon awareness
c	Blockchain application cost per unit of product
t	Retailer’s social responsibility level
p	Retail price
$p_{e}$	Carbon trading price
e	Initial carbon emission level per unit of product, 0<x<e
Decision variables	
w	Wholesale price
q	Production quantity
x	Carbon abatement level per unit product
λ	Cost-sharing ratio

## 4. Model framework

### 4.1. Basic model

In this section, the manufacturer does not adopt blockchain technology and the unit abatement cost is fully borne by the manufacturer. Both firms act with the goal of maximizing their own profit or utility. The objective functions of the two firms are:


$$\overset{N}{\underset{m}{\pi }}=wq-\left[\left(e-x\right)(a-p+\eta_{\text{1}}bx)-E]p_{e}-hx^{\text{2}}/2\right],$$ (1)

$$\overset{N}{\underset{r}{U}}=(p-w)(a-p+\eta_{\text{1}}bx)+tq^{\text{2}}/2.$$ (2)

Backward induction is applied to solve the objective functions of the two firms and obtain the equilibrium solutions in Theorem 1.

**Theorem 1.** The basic model can obtain the following optimal strategies:

$x^{N*}=\frac{\left(a-ep_{e})(p_{e}+b\eta_{\text{1}}\right)}{2h(2-t)-(p_{e}+b\eta_{\text{1}}{)}^{\text{2}}}$, $w^{N*}=\frac{h(2-t)(ep_{e}+a)-p_{e}(p_{e}+b\eta_{\text{1}})(a+be\eta_{\text{1}})}{2h(2-t)-(p_{e}+b\eta_{\text{1}}{)}^{\text{2}}}$, $q^{N*}=\frac{h(a-ep_{e})}{2h(2-t)-(p_{e}+b\eta_{\text{1}}{)}^{\text{2}}}$, $\overset{N*}{\underset{m}{\pi }}=\frac{h(a-ep_{e}{)}^{\text{2}}}{2[2h(2-t)-(p_{e}+b\eta_{\text{1}}{)}^{\text{2}}]}+Ep_{e}$, $\overset{N*}{\underset{r}{\pi }}=\frac{h^{\text{2}}(a-ep_{e}{)}^{\text{2}}(1-t)}{[2h(2-t)-(p_{e}+b\eta_{\text{1}}{)}^{\text{2}}{]}^{\text{2}}}$, $\overset{N*}{\underset{r}{U}}=\frac{h^{\text{2}}(a-ep_{e}{)}^{\text{2}}(2-t)}{2[2h(2-t)-(p_{e}+b\eta_{\text{1}}{)}^{\text{2}}{]}^{\text{2}}}$.

According to Theorem 1, the following propositions are proposed to explore the effects of relevant factors on equilibrium solutions.

**Proposition 1.** (1) $\frac{\partial x^{N*}}{\partial t}>0$; $\frac{\partial x^{N*}}{\partial \eta }<0$. (2) $\frac{\partial x^{N*}}{\partial p_{e}}>0$; $x^{N*}>{x^{N*}|}_{p_{e}=0}>0$.

Proposition 1 (1) indicates that the optimal unit carbon abatement level increases with the level of retailer social responsibility but decreases with the level of consumer skepticism. On the one hand, as retailers’ social responsibility levels increase, their concern for consumer surplus grows. Meanwhile, for environmentally conscious consumers, purchasing products with higher carbon abatement levels generates greater utility, thereby increasing both market demand and consumer surplus. Therefore, socially responsible retailers can indirectly guide manufacturers to reduce emissions by stimulating market demand—for example, by promoting low-carbon products to consumers. However, consumer skepticism diminishes the unit carbon reduction level, as such skepticism hinders product premium pricing or demand expansion—both of which are key incentives for manufacturers to engage in carbon reduction efforts.

Proposition 1 (2) reflects that an increase in the carbon price can incentivize firms to raise unit abatement levels. This is because a higher carbon price increases either the manufacturer’s emission costs or the potential gains from trading carbon credits. This finding suggests that the CTP can play a positive role in encouraging firms to reduce emissions. Furthermore, a carbon price of zero implies the absence of low-carbon regulatory intervention or a failure of the carbon market. In such cases, the manufacturer’s motivation for carbon abatement stems solely from its environmental awareness and consumers’ environmental preferences. Although the manufacturer shows willingness and takes action to reduce emissions under this condition, the optimal unit abatement level remains significantly lower than in scenarios where a CTP is in place. In other words, the CTP can serve as a positive moderating factor in corporate carbon reduction efforts and represent a critical driving force for accelerating firms’ low-carbon transitions.

**Proposition 2.** (1) $\frac{\partial \overset{N*}{\underset{m}{\pi }}}{\partial t}>0$; $\frac{\partial \overset{N*}{\underset{r}{\pi }}}{\partial t}>0$, if $0<t<\frac{(b\eta_{\text{1}}+p_{e}{)}^{\text{2}}}{2h}$; otherwise, $\frac{\partial \overset{N*}{\underset{r}{\pi }}}{\partial t}>0$. (2) $\frac{\partial \overset{N*}{\underset{m}{\pi }}}{\partial p_{e}}>0$; $\frac{\partial \overset{N*}{\underset{r}{\pi }}}{\partial p_{e}}>0$. (3) $\frac{\partial \overset{N*}{\underset{m}{\pi }}}{\partial \eta }<0$; $\frac{\partial \overset{N*}{\underset{r}{\pi }}}{\partial \eta }<0$.

Proposition 2 (1) shows that the manufacturer’s optimal profit in model N increases with the level of retailer social responsibility. This occurs because higher social responsibility level leads to greater unit abatement level and stronger market demand. On one hand, manufacturers benefit from increased carbon credit trading revenue; on the other hand, they gain additional profit from expanded demand. For retailers, moderate corporate social responsibility enhances profits; however, beyond a certain threshold, further increases reduce retailer profits. The reason lies in the fact that a moderate level of retailer social responsibility enhances consumer trust, stimulates demand, and thereby increases the profits of both firms. However, beyond a certain threshold, the marginal benefits of social responsibility gradually diminish while the associated costs rise. Excessive social responsibility may even trigger consumer skepticism, ultimately reducing the overall profitability of the supply chain. This threshold is positively associated with the impact of abatement on demand and the carbon price, but negatively associated with consumer skepticism. In other words, stronger consumer low-carbon preferences or higher carbon prices incentivize retailers to engage more actively in socially responsible behavior.

Proposition 2 (2) indicates that as the carbon price increases, the optimal profits of both manufacturers and retailers rise. A higher carbon price not only incentivizes firms to enhance their abatement levels but also amplifies consumer preferences and demand for low-carbon products, thereby allowing manufacturers and retailers to capture additional revenues through expanded sales or higher pricing. This finding suggests that firms should flexibly adjust their abatement investments in response to carbon price dynamics to achieve a win–win outcome of economic gains and environmental benefits.

Proposition 2 (3) shows that in model N, manufacturer and retailer profits decline as consumer skepticism rises. A reduction in consumer trust toward products lowers their willingness to pay and reduces demand, leading to a simultaneous decrease in profits for manufacturers and retailers. This highlights that firms cannot rely solely on carbon prices to maximize profits and should enhance consumer trust through measures such as third-party certification or blockchain-based transparency.

### 4.2. Blockchain model

To alleviate consumer skepticism about the authenticity of abatement information, this section considers a scenario in which the manufacturer introduces blockchain technology. Both abatement and blockchain application costs are borne by the manufacturer. In this case, the manufacturer and the retailer still act with the goal of maximizing their own profit or utility. The objective functions of the two firms are:


$$\overset{B}{\underset{m}{\pi }}=(w-c)(a-p+bx)-\left[\left(e-x\right)(a-p+bx)-E\right]p_{e}-hx^{\text{2}}/2,$$ (3)

$$\overset{B}{\underset{r}{U}}=(p-w)(a-p+bx)+t(a-p+bx{)}^{\text{2}}/2.$$ (4)

Backward induction is applied to solve the objective functions of the two firms and obtain the equilibrium solutions in Theorem 2.

**Theorem 2.** The blockchain model can obtain the following optimal strategies:

$x^{B*}=\frac{\left(b+p_{e})(a-c-ep_{e}\right)}{2h(2-t)-(b+p_{e}{)}^{\text{2}}}$, $w^{B*}=\frac{h(a+ep_{e}+c)(2-t)-[p_{e}(a+be)+bc](b+p_{e})}{2h(2-t)-(b+p_{e}{)}^{\text{2}}}$, $q^{B*}=\frac{h(a-c-ep_{e})}{2h(2-t)-(b+p_{e}{)}^{\text{2}}}$, $\overset{B*}{\underset{m}{\pi }}=\frac{h(a-c-ep_{e}{)}^{\text{2}}}{2[2h(2-t)-(b+p_{e}{)}^{\text{2}}]}+Ep_{e}$, $\overset{B*}{\underset{r}{\pi }}=\frac{h^{\text{2}}(a-c-ep_{e}{)}^{\text{2}}(1-t)}{[2h(2-t)-(b+p_{e}{)}^{\text{2}}{]}^{\text{2}}}$, $\overset{B*}{\underset{r}{U}}=\frac{h^{\text{2}}(a-c-ep_{e}{)}^{\text{2}}(2-t)}{2[2h(2-t)-(b+p_{e}{)}^{\text{2}}{]}^{\text{2}}}$.

According to Theorem 2, the following propositions are proposed to explore the effects of relevant factors on equilibrium solutions.

**Proposition 3.** (1) $\frac{\partial x^{B*}}{\partial c}<0$. (2) $\frac{\partial \overset{B*}{\underset{m}{\pi }}}{\partial c}<0$; $\frac{\partial \overset{B*}{\underset{r}{\pi }}}{\partial c}<0$.

Proposition 3 (1) demonstrates that the cost of blockchain adoption leads to a reduction in the unit abatement level. This is because blockchain adoption increases firms’ cost burden while reducing information asymmetry through emission data transparency. Consequently, the return on carbon reduction investments increases, while the marginal reputational benefit of ‘excessive abatement’ declines, prompting firms to lower the unit carbon abatement level to a more economically rational point.

Proposition 3 (2) demonstrates that the optimal profit of both the manufacturer and the retailer decrease as the cost of blockchain application increases. While blockchain improves supply chain transparency and consumer trust, its high implementation cost can offset these benefits by reducing firms’ profitability. Thus, effective cost-sharing mechanism is essential to balance the gains in credibility against the financial burden. For instance, Walmart’s adoption of IBM’s Food Trust blockchain greatly improved product transparency and consumer trust. Yet, the high upfront costs of system development, maintenance, and training eroded profits and discouraged participation by smaller suppliers.

**Proposition 4.** $x^{B*}>x^{N*}$, if *c* < *c*_1_; otherwise, $x^{N*}>x^{B*}$. Here, $c_{\text{1}}=A[1-\frac{B_{\text{1}}(hT-{B_{\text{2}}}^{\text{2}})}{B_{\text{2}}(hT-{B_{\text{1}}}^{\text{2}})}]$, $A=a-ep_{e}$, $B_{\text{1}}=p_{e}+b\eta_{\text{1}}$, $B_{\text{2}}=b+p_{e}$, T=2(2−t).

Proposition 4 indicates that, under certain conditions ($c<c_{\text{1}}$), the unit abatement level with blockchain adoption can be higher than in the absence of blockchain. The application of blockchain reduces consumer skepticism, thereby increasing the market demand for low-carbon products. Constrained by the CTP, firms must lower the carbon emissions per product to control total emissions. Consequently, when the unit cost of blockchain adoption is below a certain threshold, manufacturers should implement blockchain. However, if the cost exceeds this threshold, product prices rise and market demand declines. In this case, blockchain adoption may reduce the unit abatement level. Moreover, the threshold is positively correlated with market size and the degree of consumer skepticism, indicating that industries with larger markets or higher consumer skepticism have a natural advantage in using blockchain for verifying carbon reduction information. For example, Starbucks has implemented blockchain to monitor carbon emissions across its supply chain, achieving both cost reduction and emission mitigation through smart energy management and green electricity procurement. In contrast, smaller coffee brands often cannot afford such complex systems.

**Proposition 5.** (1) $\overset{B*}{\underset{m}{\pi }}>\overset{N*}{\underset{m}{\pi }}$, if $c<c_{\text{2}}$; otherwise, $\overset{B*}{\underset{m}{\pi }}<\overset{N*}{\underset{m}{\pi }}$. (2) $\overset{B*}{\underset{r}{\pi }}>\overset{N*}{\underset{r}{\pi }}$, if $c<c_{\text{3}}$; otherwise, $\overset{B*}{\underset{r}{\pi }}<\overset{N*}{\underset{r}{\pi }}$. (3) $\overset{B*}{\underset{m}{\pi }}>\overset{N*}{\underset{m}{\pi }}$ and $\overset{B*}{\underset{r}{\pi }}>\overset{N*}{\underset{r}{\pi }}$, if $c<c_{\text{2}}$.

Here, $c_{\text{2}}=A[1-\sqrt{\left(hT-{B_{\text{2}}}^{\text{2}})/(hT-{B_{\text{1}}}^{\text{2}}\right)}]$, $c_{\text{3}}=A[1-(hT-{B_{\text{2}}}^{\text{2}})/(hT-{B_{\text{1}}}^{\text{2}})]$, $A=a-ep_{e}$, $B_{\text{1}}=p_{e}+b\eta_{\text{1}}$, $B_{\text{2}}=b+p_{e}$, T=2(2−t).

Proposition 5 (1) indicates that, compared to the scenario without blockchain, manufacturers can achieve higher profits under blockchain scenario when the application cost is below a certain threshold ($c_{\text{2}}$). (2) When the blockchain application cost is below $c_{\text{3}}$, blockchain adoption by manufacturer improves retailer profits. Blockchain reduces consumer skepticism and thus has a positive impact on market demand and unit abatement levels. Therefore, when the sales profit from market expansion and the carbon credit benefit from abatement can jointly offset the cost of blockchain application, the manufacturer should adopt blockchain.

Proposition 5 (3) states that when the application cost of blockchain is below a certain threshold ($c<c_{\text{2}}$), the two firms can achieve a win–win situation in terms of profit. The adoption of blockchain reduces the manufacturer’s investment in abatement. However, when the cost of blockchain is low, the impact of blockchain on abatement is small, and blockchain is also conducive to increasing market demand. Therefore, when the contribution of blockchain in increasing demand outweighs the negative impact of reduced investment in abatement, both firms can obtain higher profits. This threshold is positively correlated with market size and consumer skepticism.

### 4.3. Cost-sharing extension of the blockchain model

Given that the costs of abatement and blockchain are borne by the manufacturer, this section considers the scenario where the retailer shares the cost of the manufacturer. In addition, this section gives the optimal cost-sharing ratio *λ* in two ways, namely, the retailer decision *λ* and the two-firm bargaining decision *λ*. The objective functions of the two firms are:


$$\overset{BS}{\underset{m}{\pi }}=w(a-p+bx)-\left[\left(e-x\right)(a-p+bx)-E\right]p_{e}-(1-\lambda )[hx^{\text{2}}/2+c(a-p+bx)],$$ (5)

$$\overset{BS}{\underset{r}{U}}=(p-w)(a-p+bx)+t(a-p+bx{)}^{\text{2}}/2-\lambda [hx^{\text{2}}/2+c(a-p+bx)].$$ (6)

Backward induction is applied to solve the objective functions of the two firms and obtain the equilibrium solutions in Theorem 3.

**Theorem 3.** The cost-sharing model can obtain the following optimal strategies:

$x^{BS*}=\frac{\left(b+p_{e})(a-c-ep_{e}\right)}{2h(1-\lambda )(2-t)-(b+p_{e}{)}^{\text{2}}}$, $w^{BS*}=\frac{\begin{array}{c} h(a+ep_{e}+c)(1-\lambda )(2-t)\\ -[p_{e}(a+be)+bc](b+p_{e}) \end{array}}{2h(1-\lambda )(2-t)-(b+p_{e}{)}^{\text{2}}}-c\lambda$, $q^{BS*}=\frac{h(1-\lambda )(a-c-ep_{e})}{2h(1-\lambda )(2-t)-(b+p_{e}{)}^{\text{2}}}$, $\overset{BS*}{\underset{m}{\pi }}=\frac{h(a-c-ep_{e}{)}^{\text{2}}(1-\lambda )}{2[2h\left(1-\lambda \right)\left(2-t\right)-(b+p_{e}{)}^{\text{2}}]}+Ep_{e}$, $\overset{BS*}{\underset{r}{\pi }}=\begin{array}{c} h{\left(a-c-ep_{e}\right)}^{2}\\ \frac{\left[2h(1-t){\left(1-\lambda \right)}^{2}-\lambda {\left(b+p_{e}\right)}^{2}\right]}{2{\left[2h(1-\lambda )(2-t)-{\left(b+p_{e}\right)}^{2}\right]}^{2}} \end{array}$, $\overset{BS*}{\underset{r}{U}}=\begin{array}{c} h(a-c-ep_{e}{)}^{\text{2}}\\ \frac{\left[h\left(2-t\right)(1-\lambda {)}^{\text{2}}-\lambda (b+p_{e}{)}^{\text{2}}\right]}{2{\left[2h(1-\lambda )(2-t)-(b+p_{e}{)}^{\text{2}}\right]}^{\text{2}}} \end{array}$.

According to Theorem 3, the following propositions are proposed to explore the effects of relevant factors on different models.

**Proposition 6.** (1) $\frac{\partial x^{BS*}}{\partial \lambda }>0$. (2) $\frac{\partial \overset{BS*}{\underset{r}{\pi }}}{\partial \lambda }>0$ if $\lambda <\frac{(b+p_{e}{)}^{\text{2}}-2ht}{h(8-6t)}$; otherwise, $\frac{\partial \overset{BS*}{\underset{r}{\pi }}}{\partial \lambda }<0$.

Proposition 6 (1) demonstrates that the manufacturer’s optimal profit is positively related to the cost-sharing ratio in the BS model. On one hand, manufacturers directly benefit from cost-sharing. On the other hand, the retailer’s participation in cost-sharing contributes to an increase in the unit carbon abatement level. Given consumers’ preference for low-carbon products, a higher unit abatement level leads to increased sales profits for manufacturers. Additionally, a higher reduction level enhances the manufacturer’s revenue from carbon credit trading.

Proposition 6 (2) shows that when the share of costs borne by the retailer is less than a certain threshold, the retailer can obtain higher profits through cost-sharing, thus achieving a win–win situation with the manufacturer. Under the premise that the manufacturer invests in abatement and blockchain, the retailer can obtain high profits by “free riding”. After the cost-sharing ratio is greater than a certain threshold, the beneficiaries of “free riding” change from the retailer to the manufacturer. In addition, the threshold is positively correlated with the impact factor of abatement on demand and the carbon price. This correlation indicates that the retailer can share the higher cost when the carbon price or the impact factor of abatement on demand is high.

**Proposition 7.** (1) $x^{BS*}>x^{N*}$, if $c<c_{\text{4}}$; otherwise, $x^{BS*}<x^{N*}$. (2) $\overset{BS*}{\underset{m}{\pi }}>\overset{N*}{\underset{m}{\pi }}$, if 0 < *c* < *c*_5_; otherwise, $\overset{BS*}{\underset{m}{\pi }}<\overset{N*}{\underset{m}{\pi }}$. (3) $\overset{BS*}{\underset{r}{\pi }}>\overset{N*}{\underset{r}{\pi }}$, if 0 < *c* < *c*_6_; otherwise, $\overset{BS*}{\underset{r}{\pi }}<\overset{N*}{\underset{r}{\pi }}$. (4) $\overset{BS*}{\underset{m}{\pi }}>\overset{N*}{\underset{m}{\pi }}$ and $\overset{BS*}{\underset{r}{\pi }}>\overset{N*}{\underset{r}{\pi }}$, if 0 < *c* < min {*c*_5_, *c*_6_}. Here, $c_{\text{4}}=A[1-\frac{B_{\text{1}}[(1-\lambda )hT-{B_{\text{2}}}^{\text{2}}]}{B_{\text{2}}(hT-{B_{\text{1}}}^{\text{2}})}]$, $c_{\text{5}}=A[1-\sqrt{\frac{\lambda_{\text{1}}hT-{B_{\text{2}}}^{\text{2}}}{\lambda_{\text{1}}(hT-{B_{\text{1}}}^{\text{2}})}}]$, $c_{\text{6}}=A[1-\frac{\lambda_{\text{1}}hT-{B_{\text{2}}}^{\text{2}}}{hT-{B_{\text{1}}}^{\text{2}}}\sqrt{\frac{h(T-2)}{h(T-2){\lambda_{\text{1}}}^{\text{2}}-(1-\lambda_{\text{1}}){B_{\text{1}}}^{\text{2}}}}]$, $A=a-ep_{e}$, $B_{\text{1}}=p_{e}+b\eta_{\text{1}}$, $B_{\text{2}}=b+p_{e}$, T=2(2−t), *λ*_1_ = 1−*λ*.

Proposition 7 shows that when the cost of blockchain application is below a certain threshold ($c<c_{\text{4}}$), the manufacturer can achieve a higher unit abatement level in the BS model; when the cost of blockchain application exceeds this threshold, the manufacturer can achieve a higher unit abatement level in the N model. The threshold is positively influenced by the cost-sharing ratio. This outcome suggests that the cost-sharing behavior of the retailer can reduce the threshold for blockchain adoption by the manufacturer.

In addition, the manufacturer or retailer becomes more profitable under the BS model than under the N model when the unit application cost of blockchain is below a certain threshold ($0<c<c_{\text{5}}$ or $0<c<c_{\text{6}}$), respectively. Moreover, when the unit application cost of blockchain is below a certain threshold ($0<c<min\left\{c_{\text{5}},c_{\text{6}}\right\}$), both firms can obtain higher profits in the BS model than in the N model. The main reason is that an appropriate cost-sharing ratio by the retailer effectively motivates the manufacturer to adopt blockchain technology and enhance carbon abatement efforts, which in turn stimulates market demand and increases the profits of both firms.

#### 4.3.1. Retailer decides the cost-sharing ratio.

This section examines the retailer’s optimal cost-sharing strategy when the sharing ratio is endogenously determined [6]. By substituting the equilibrium strategies from Theorem 3 into the utility function, the following corollary can be derived:

**Corollary 1.** The optimal endogenous cost-sharing ratio in the R model is $\lambda^{R*}=\frac{(b+p_{e}{)}^{\text{2}}}{4h(2-t)}$.

Corollary 1 shows that the optimal cost-sharing ratio is positively correlated with the level of consumer low-carbon preference, carbon price, and social responsibility level. With the increase of consumers’ low-carbon preference level or carbon price, carbon abatement can bring higher sales profits to the two firms, and the manufacturer can obtain higher potential carbon credit trading income. In this case, either the manufacturer or the retailer is willing to increase the level of abatement. Therefore, the retailer is willing to bear a higher proportion of the cost. Moreover, with the improvement of social responsibility level, the retailer’s attention to consumer surplus increases. To increase consumer surplus, the retailer must reduce retail prices or increase product abatement levels, making the retailer willing to share more of the costs. For example, to meet consumers’ low-carbon expectations and fulfill its social responsibility, Starbucks actively collaborates with suppliers to share the investment costs of green technologies, such as funding coffee farmers to adopt energy-efficient roasting equipment or renewable energy facilities. This practice aligns with corollary 1, demonstrating how a retailer’s cost-sharing willingness can incentivize manufacturers to reduce emissions.

#### 4.3.2. Nash bargains.

Companies usually bargain to determine the cost-sharing ratio ([46,47]). Based on $\overset{BS*}{\underset{m}{\pi }}$ and $\overset{BS*}{\underset{r}{U}}$ in Theorem 3, this study constructs a Nash bargaining model (NB model). In reference to Palit and Brint [56], the model is set to $\underset{\lambda }{max}\;\Pi^{NB}\left(\lambda \right)=\overset{BS*}{\underset{m}{\pi }}*\overset{BS*}{\underset{r}{U}}$. By solving this model, the following corollary can be obtained:

**Corollary 2.** The optimal endogenous cost-sharing ratio in the NB model is $\lambda^{NB*}=(1-\overset{*}{\underset{\text{1}}{\lambda }})$. Here, $\overset{*}{\underset{\text{1}}{\lambda }}=\frac{[2GI(4Ih+H)+F(2Ih-H)]+\sqrt{\left[2GI(4Ih+H)+F(2Ih-H){]}^{\text{2}}+(16GI^{\text{2}}+5FI)(2GH^{\text{2}}+FHh-8GIHh\right)}}{(16GI+5F)Ih}$, $F=(a-c-ep_{e}{)}^{\text{2}}$, $G=Ep_{e}$, $H=(b+p_{e}{)}^{\text{2}}$, and I=2−t.

**Fig 2:**
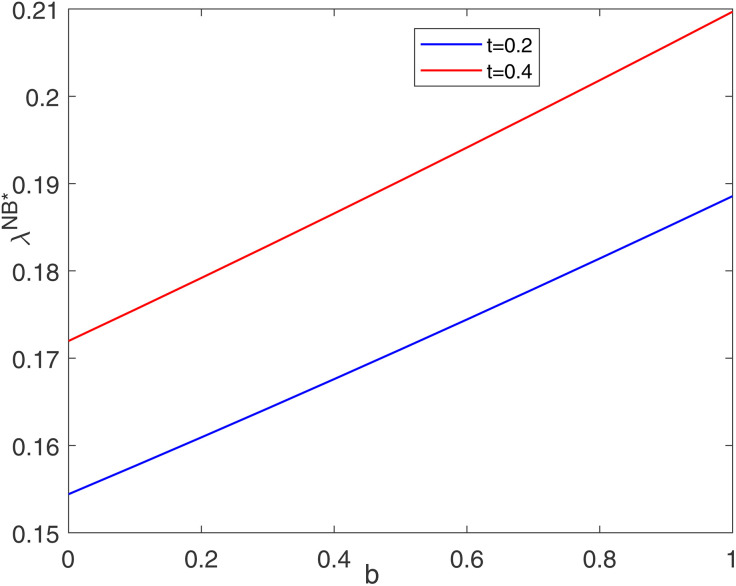
Impacts of b on optimal cost-sharing ratio.

**Fig 3:**
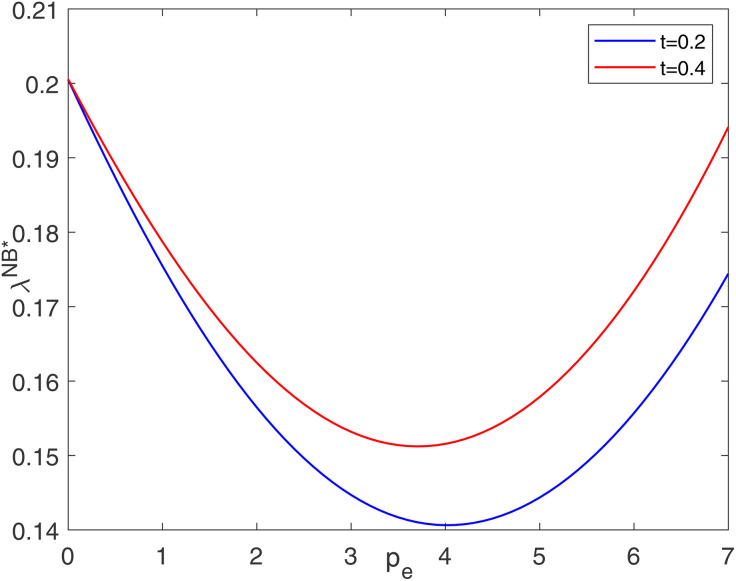
Impacts of $p_{e}$ on optimal cost-sharing ratio.

Corollary 2 shows that the optimal cost-sharing ratio is affected by consumer preference, carbon price, retailer’s social responsibility level, blockchain application cost, and manufacturer’s total emission credits. Figs 2 and 3 show the impact of some factors on the optimal cost-sharing ratio. In reference to similar studies [57], the basic parameters in this section are set to a=200, b=0.6, c=1.5, e=14, $p_{e}=7$, t=0.2, h=60, η=0.3, and E=1000.

As shown in Fig 2, the cost-sharing ratio is positively correlated with the level of consumers’ low-carbon preferences. When the level of consumers’ low-carbon preference is high, carbon abatement can greatly increase the quantity of market demand. Therefore, the retailer is willing to bear more costs. Fig 3 suggests that the optimal cost-sharing ratio has a U-shaped relationship with the carbon price. When the carbon price is at a low level, carbon abatement can generate higher profits for the manufacturer as the carbon price increases. Therefore, the retailer moderately reduces the cost-sharing ratio. However, when the carbon price is at a high level, the manufacturer increases the abatement level under the stimulation of the carbon trading profit, leading to a substantial increase in the abatement cost. In this case, the retailer moderately increases its cost-sharing ratio.

## 5. Numerical study

This section presents numerical examples to intuitively illustrate and verify the relevant conclusions of Section 4, which is a common approach in the field of operations management [24,26,57]. The selection of parameter values follows the principles outlined below: (1) Consistency with reality and model assumptions (e.g., the carbon abatement cost coefficient h should be relatively large to reflect the high investment and difficulty associated with carbon reduction in practice; the potential market size a should be large, while the unit blockchain cost c should be small, to ensure a positive marginal profit); (2) Compliance with all mathematical boundary conditions assumed in the study (e.g., λ<(1−t)/(3−2t), as in reality the cost-sharing proportion among firms is expected to be low and well below 1); (3) The parameter values are common values, and changes without violating basic common sense and research assumptions will not change the main conclusions. Based on the above principles and existing studies [58,59], the parameter values in this section are set as a=200, b=0.6, c=1.5, e=14, $p_{e}=7$, t=0.2, h=60, η=0.3, and E=1000.

### 5.1. Effects of blockchain technology

**Fig 4:**
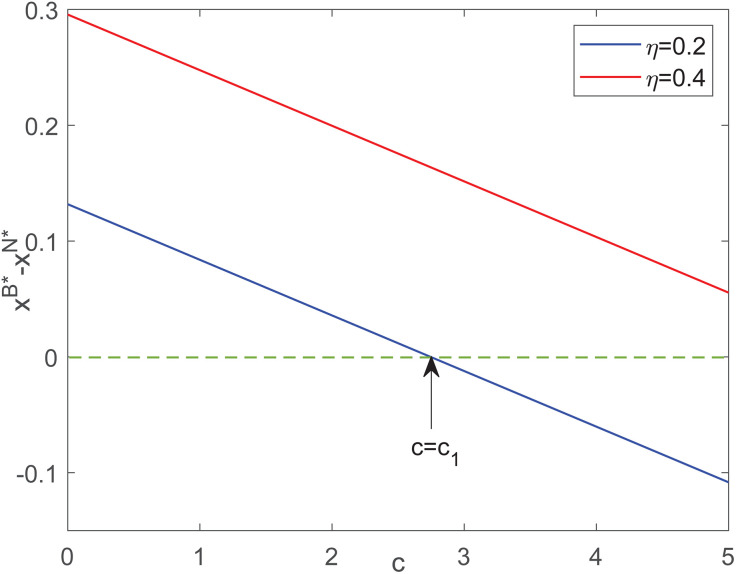
Impacts of c on optimal abatement level gap between different models.

Fig 4 indicates that the advantage of B model in abatement decreases as the blockchain application cost increases. When $c>c_{\text{1}}$, the introduction of blockchain technology by the manufacturer results in a lower unit abatement level in model B than in model N. In addition, from the perspective of unit abatement level, the increase in consumer skepticism can increase the advantage of model B. This suggests that high-emission firms should conduct a marginal analysis of carbon reduction benefits versus technology adoption costs before introducing blockchain technologies, ensuring that such investments do not crowd out core environmental initiatives.

**Fig 5:**
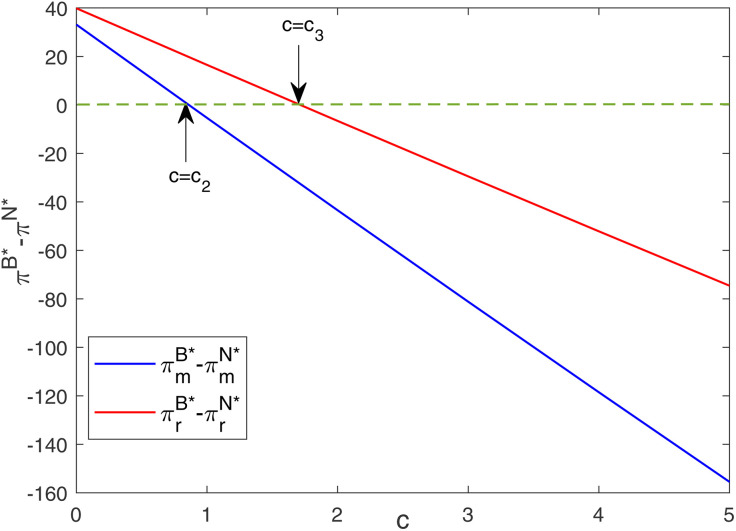
Impacts of c on optimal profit gap between different models.

Fig 5 illustrates the attractiveness of blockchain technology to both firms decrease with the increase in blockchain cost per unit of product. Fig 5 also verifies proposition 5. That is, when the unit cost of adopting blockchain is lower than a certain threshold ($c<c_{\text{2}}$), the introduction of blockchain can increase the profits of the two firms. When the unit cost of adopting blockchain exceeds a certain threshold ($c>c_{\text{3}}$), both firms refuse to adopt blockchain. This result also offers meaningful policy implications for governments aiming to promote low-carbon digital technologies. Specifically, whether blockchain technology is favored at the firm level fundamentally depends on its economic feasibility.

### 5.2. Analysis of the cost-sharing contract

**Fig 6:**
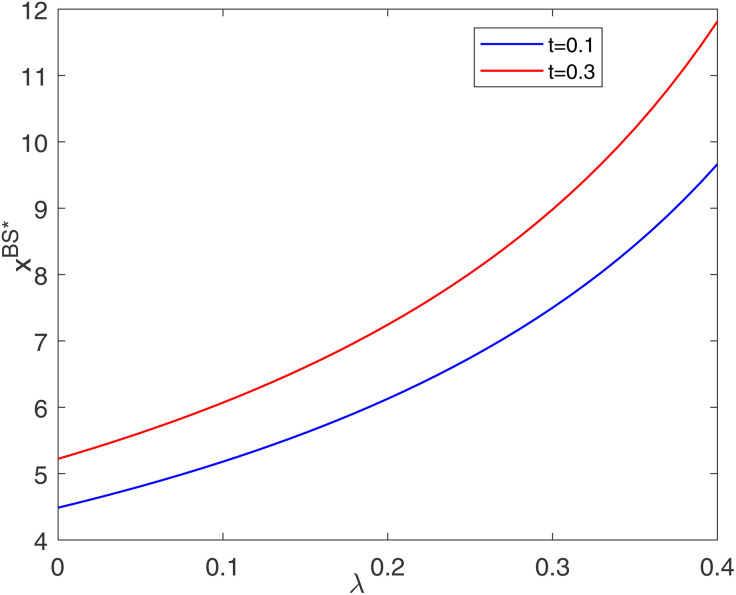
Impacts of λ on optimal abatement level.

Fig 6 shows that the optimal unit carbon abatement level in the BS model is positively correlated with the cost-sharing ratio, and the higher the level of social responsibility, the more significant the contribution of the cost-sharing ratio to the unit carbon abatement level. On the one hand, an increase in the social responsibility level can directly increase the unit abatement level. On the other hand, an increase in the social responsibility level of the retailer increases its cost-sharing ratio, which in turn leads the manufacturer to be able to invest more funds in carbon abatement.

**Fig 7:**
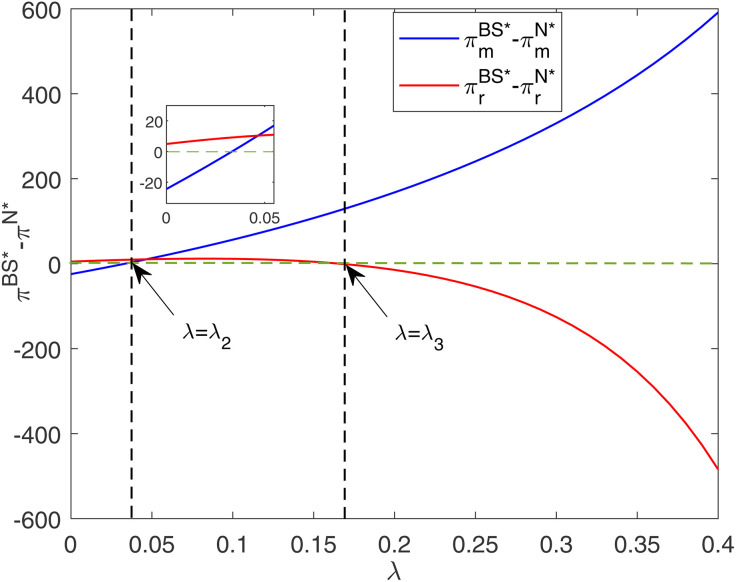
Impacts of λ on optimal profit gap between different models.

Fig 7 indicates that the manufacturer’s optimal profit gap between the BS and N models increases as the cost-sharing ratio increases. Similarly, the retailer’s optimal profit gap between the BS and N models shows an inverted U-shaped relationship. This relationship shows that the moderate cost sharing by the retailer can increase the level of unit reduction and market demand, thereby benefiting both firms. However, when the cost-sharing ratio is at a high level, the additional profit gained by the retailer will not be sufficient to offset the cost it shares. In this scenario, the retailer’s profit becomes lower in the BS model than in the N model. Under certain conditions ($\lambda_{\text{2}}<\lambda <\lambda_{\text{3}}$), the cost-sharing contract can improve the profits of both firms.

### 5.3. Effects of carbon price and consumer skepticism

**Fig 8:**
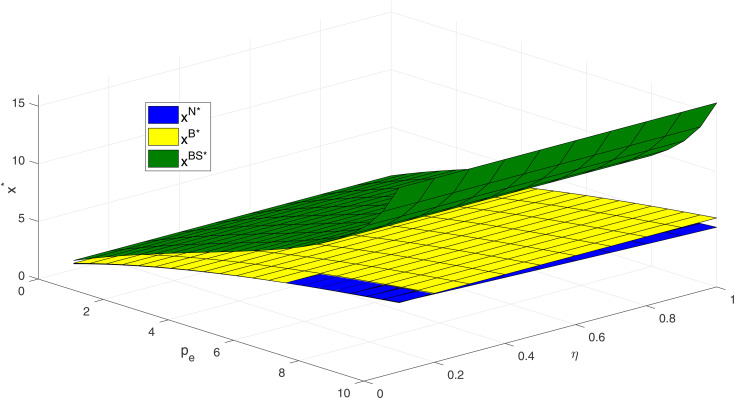
Impacts of $p_{e}$ and η on optimal abatement level.

Fig 8 shows the impact of carbon price and consumer skepticism on optimal carbon abatement level under different models. The results show that as carbon price increases, unit carbon abatement levels rise across all three models, indicating that higher carbon prices strengthen firms’ economic incentives for abatement. In the non-blockchain scenario, unit abatement level remains low, and at a constant carbon price, the unit abatement level decreases as consumer skepticism rises, indicating that in the absence of credible mechanisms, consumer skepticism provides little additional incentive for firms to reduce emissions. In contrast, in the scenario combining blockchain and cost-sharing mechanisms, unit abatement level is highest and increases with carbon price. This demonstrates that the combination of blockchain and cost-sharing not only enhances consumer trust by improving abatement credibility but also reduces firms’ cost pressure, thereby stimulating stronger abatement incentives and highlighting the superimposed effect of these mechanisms.

**Fig 9:**
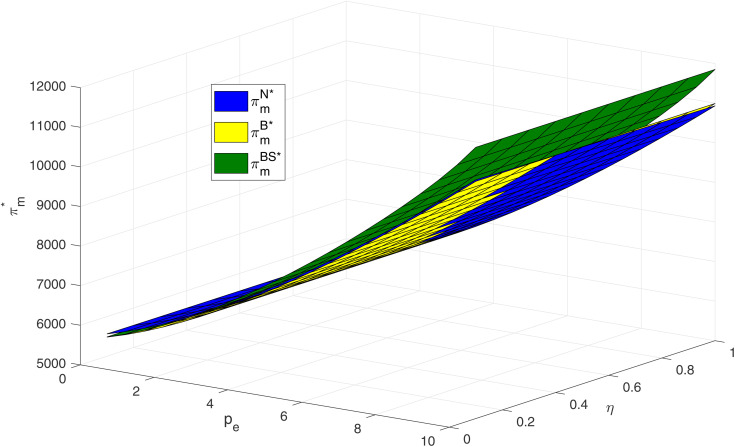
Impacts of $p_{e}$ and η on manufacturer profit.

As shown in Fig 9, within the numerical range considered, manufacturer profits increase with carbon price across all models. This may be attributed to higher carbon prices enhancing the economic value of abatement (via increased carbon trading revenue) or to consumers’ greater willingness to pay for low-carbon products. In the N model, however, consumer skepticism reduces firm profitability, indirectly underscoring the economic value of blockchain. When both carbon price and consumer skepticism are low, manufacturers achieve higher profits in the N model compared to the S and BS models, partially corroborating the findings of Propositions 5 and 7. Overall, from the perspective of manufacturer profit, the BS model is superior, followed by the S model. These results suggest that firms should closely monitor carbon market dynamics, adopt blockchain strategically in response to consumer skepticism, and carefully balance implementation costs against expected profit gains.

**Fig 10:**
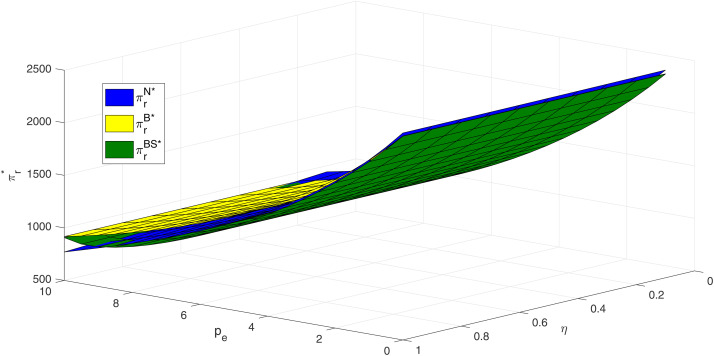
Impacts of $p_{e}$ and η on retailer profit.

Fig 10 indicates that, within the numerical range considered, retailer profits increase with carbon price across all models. When carbon prices are high and consumer skepticism is low, the N model yields higher profits for retailers. However, at high carbon prices, as consumer skepticism increases, retailer profits in the N model gradually fall below those in the S model. Overall, only when both carbon price and consumer skepticism are high does the BS model yield profits exceeding those of the N model. These results suggest that in contexts characterized by high consumer skepticism—particularly under elevated carbon prices—retailers should proactively support manufacturers in adopting blockchain technology to safeguard their profitability.

## 6. Concluding remarks

In the context of CTP, this study first investigates how consumer skepticism regarding product abatement levels influences the operational and abatement strategies of corporates in a supply chain consisting of a manufacturer and a socially responsible retailer. To mitigate consumer skepticism, the study then considers the application of blockchain technology by the manufacturer and analyzes the impact of blockchain on decisions. Furthermore, cost-sharing contracts are introduced to improve coordination and maximize profits for both parties. The optimal cost-sharing ratios are provided under the retailer decision strategy and the Nash bargaining decision strategy.

### 6.1. Conclusions

This study yields several important conclusions. First, greater consumer skepticism reduces manufacturers’ abatement levels and joint profits, highlighting the importance of accounting for behavioral factors often overlooked in supply chain abatement models. By contrast, stronger consumer low-carbon preferences and higher carbon prices incentivize socially responsible retailer behavior, thereby increasing the profits of each member.

Second, unlike Zhou et al. [34], who suggest that retailers can always benefit from blockchain-driven information disclosure, our study finds that firms can only gain when blockchain costs are below a certain threshold. This finding extends the research on blockchain in information verification by revealing how market and behavioral conditions jointly determine its feasibility as a credibility-enhancing mechanism. This further implies that, prior to blockchain deployment, firms should avoid indiscriminate investment and instead systematically assess key factors—such as cost structures, market conditions, and consumer behavior—to ensure optimal resource allocation.

Third, Cost-sharing contracts enhance manufacturers’ incentives to reduce emissions, expand market demand, and improve profits, while retailers’ profits have an inverted U-shaped relationship with the cost-sharing ratio. This finding differs from Fang et al. [38], who suggest that cost-sharing contracts enable manufacturers and retailers to achieve higher profits. Moreover, our results show that higher carbon prices, stronger consumer low-carbon preferences, and greater social responsibility increase retailers’ willingness to share costs, highlighting how behavioral and market factors interact with contractual design.

Finally, the derivation of optimal sharing ratios under retailer decision-making and Nash bargaining frameworks enriches coordination theory by revealing distinct effects to carbon prices, carbon caps, social responsibility, and consumer preference. These insights advance the development of coordination theory in low-carbon supply chains by linking carbon abatement, consumer behavior, and cooperative contracts.

### 6.2. Managerial insights

Based on the aforementioned findings, several key management insights emerge. Manufacturers should ensure transparency and credibility when disclosing product carbon abatement information, moving beyond the traditional “focus on technology, neglect consumer awareness” approach. Elevating consumer recognition should be central, achieved through trustworthy information disclosure and transparent carbon reduction processes, as any misrepresentation could undermine both their own and their partners’ profitability. A salient example is Volkswagen’s 2015 emissions scandal, which resulted in over $20 billion in fines and settlements [35]. When blockchain adoption costs are relatively low, manufacturers are advised to implement blockchain-based information systems to enhance credibility. To incentivize retailer participation in cost-sharing, manufacturers can employ mechanisms such as wholesale price concessions, particularly under high carbon price conditions.

Retailers can leverage consumers’ low-carbon preferences and abatement policies to optimize their low-carbon product portfolios, implement differentiated marketing strategies, and proactively fulfill social responsibility, thereby maintaining optimal engagement in environmentally friendly practices. Moreover, retailers should align their cost-sharing decisions with manufacturers’ blockchain strategies and take a proactive role in sharing the associated costs, especially in markets where consumer low-carbon preferences are strong. Coordinating carbon reduction through cost-sharing contracts can maximize joint profitability, and the retailer’s sharing ratio should increase correspondingly when carbon prices rise or consumer environmental awareness strengthens.

Governments should design carbon quota allocation and carbon pricing mechanisms that reflect environmental and market realities. Additionally, governments can leverage blockchain technology to establish standardized platforms for carbon abatement disclosure at industry or national levels, ensuring data transparency and consistency, and mitigating consumer skepticism. A practical precedent is the June 2021 Guiding Opinions issued by China’s Ministry of Industry and Information Technology and the Office of the Central Cyberspace Affairs Commission, which advocates blockchain-based supply chain platforms and multi-party credit systems to enhance industrial integrity. Moreover, policymakers can develop targeted support measures for blockchain applications—such as subsidies for low-cost blockchain systems—and promote the establishment of industry-wide low-carbon cost-sharing mechanisms, thereby facilitating the sustainable development of supply chains and the responsible adoption of emerging technologies.

Consumers play a pivotal role in driving low-carbon practices. They should cultivate the ability to discern genuine carbon reductions from greenwashing claims, understanding the intrinsic value of low-carbon products within the broader context of environmental preservation. Meanwhile, they should leverage market-based purchasing behavior to create demand-driven pressure, directly eliminating false low-carbon products and compelling firms to invest in substantive carbon abatement technologies and credible information disclosure, rather than superficial green marketing. In addition, consumers’ environmental awareness and rational skepticism can be transformed into a persistent market-based oversight force, filling gaps in administrative regulation and creating synergistic governance alongside policy measures.

### 6.3. Limitations and future research

This study still has some limitations. First, it only considers a single firm’s investment in abatement and blockchain, whereas future studies could explore blockchain investment strategies across multiple supply chain firms. Second, we employ a deterministic linear demand function to describe potential market demand; future research could consider demand uncertainty. Additionally, this study focuses on the effects of the CTP, blockchain adoption, and consumer skepticism on firms’ abatement and profits. Future work could further investigate how these factors influence firms’ production and pricing decisions in greater detail.

## Supporting information

S1 Appendix Appendix. (DOCX)
