# Supplementary material for: Supply chain carbon reduction considering consumer skepticism and blockchain technology under the cap-and-trade policy
Source: PLoS One. 2026 Apr 17;21(4):e0345379. doi: 10.1371/journal.pone.0345379 (PMC13089761; doi:10.1371/journal.pone.0345379)
Supplement: S1 Appendix — (DOCX) [file pone.0345379.s001.docx]

**Appendix**

**Proof of Theorem 1**

According to $\frac{\partial U_{r}^{N}}{\partial p}=a-2p+w-t(a-p+b\eta_{1}x)+b\eta_{1}x$ and$\frac{\partial^{2}U_{r}^{N}}{\partial p^{2}}=-2+t<0$, we know that $U_{r}^{N}$ is a concave function about $p$. Let $\frac{\partial U_{r}^{N}}{\partial p}=0$; then, we can obtain the reaction function $p^{N}(x,w)=-\frac{a+w-t(a+b\eta_{1}x)+b\eta_{1}x}{t-2}$. By substituting $p^{N}(x,w)$ into $\pi_{m}^{N}$, we are able to obtain the Hessian matrix $H(w,x)=\left[\begin{matrix} 2/(t-2) & (p_{e}-b\eta_{1})/(t-2) \\ (p_{e}-b\eta_{1})/(t-2) & -(ht+2p_{e}b\eta_{1}-2h)/(t-2) \end{matrix}\right]$. Given$\frac{\partial^{2}\pi_{m}^{N}}{\partial w^{2}}<0$ and when $2h(2-t)>(p_{e}+b\eta_{1})^{2}$, . Thus, $\pi_{m}^{N}$ is jointly concave in $w$ and $x$. According to $\frac{\partial\pi_{m}^{N}}{\partial w}=0$ and $\frac{\partial\pi_{m}^{N}}{\partial x}=0$, we have $w^{N*}=\frac{p_{e}(p_{e}+b\eta_{1})(a+be\eta_{1})+h(t-2)(ep_{e}+a)}{(p_{e}+b\eta_{1})^{2}-2h(2-t)}$ and $x^{N*}=-\frac{(a-ep_{e})(p_{e}+b\eta_{1})}{(p_{e}+b\eta_{1})^{2}-2h(2-t)}$. Furthermore, we can obtain $p^{N*}=\frac{h(2at-3a-ep_{e})+p_{e}(p_{e}+b\eta_{1})(a+eb\eta_{1})}{(p_{e}+b\eta_{1})^{2}-2h(2-t)}$. Finally, we can obtain $q^{N*}$, $\pi_{m}^{N*}$, $\pi_{r}^{N*}$, and $U_{r}^{N*}$.

**Proof of Proposition 1**

(1) $\frac{\partial x^{N*}}{\partial t}=\frac{2h(a-ep_{e})(p_{e}+b\eta_{1})}{[(p_{e}+b\eta_{1})^{2}-2h(2-t)]^{2}}>0$;$\frac{\partial x^{N*}}{\partial\eta_{1}}=\frac{b(a-ep_{e})[(p_{e}+b\eta_{1})^{2}+2h(2-t)]}{[(p_{e}+b\eta_{1})^{2}-2h(2-t)]^{2}}>0$, and $\eta_{1}=1-\eta$. Thus, $\frac{\partial x^{N*}}{\partial\eta}<0$.

(2) Given $\frac{\partial x^{N*}}{\partial p_{e}}>0$, $p_{e}>0$. Thus, $x^{N*}>\left.x^{N*}\right|_{p_{e}=0}$.

**Proof of Proposition 2**

(1) $\frac{\partial\pi_{r}^{N*}}{\partial t}=\frac{h^{2}(a-ep_{e})^{2}[(b\eta_{1}+p_{e})^{2}-2ht]}{[2h(2-t)-(b\eta_{1}+p_{e})]^{3}}$. The sign of $\frac{\partial\pi_{r}^{N*}}{\partial t}$is determined by the sign of $(b\eta_{1}+p_{e})^{2}-2ht$. Thus, if $t<\frac{(b\eta_{1}+p_{e})^{2}}{2h}$, then $\frac{\partial\pi_{r}^{N*}}{\partial t}>0$; otherwise, $\frac{\partial\pi_{r}^{N*}}{\partial t}<0$.

(2)$\frac{\partial\pi_{m}^{N*}}{\partial p_{e}}=\frac{4h(b\eta_{1}+p_{e})(a+ep_{e})^{2}}{[2(b\eta_{1}+p_{e})^{2}-4h(2-t)]^{2}}+\frac{eh(a+ep_{e})}{2h(2-t)-(b\eta_{1}+p_{e})^{2}}+E>0$;$\frac{\partial\pi_{r}^{N*}}{\partial p_{e}}=\frac{2h^{2}(a+ep_{e})(1-t)[2ab\eta_{1}+p_{e}(2a+ep_{e})+2eh(2-t)-b^{2}e\eta_{1}^{2}]}{[2h(2-t)-(p_{e}+b\eta_{1})^{2}]^{3}}>0$.

(3) $\frac{\partial\pi_{m}^{N*}}{\partial\eta_{1}}=\frac{4bh(b\eta_{1}+p_{e})(a+ep_{e})^{2}}{[2(b\eta_{1}+p_{e})^{2}-4h(2-t)]^{2}}>0$, and $\eta_{1}=1-\eta$. Thus, $\frac{\partial\pi_{m}^{N*}}{\partial\eta}<0$; $\frac{\partial\pi_{r}^{N*}}{\partial\eta_{1}}=\frac{4bh^{2}(1-t)(b\eta_{1}+p_{e})(a+ep_{e})^{2}}{[2h(2-t)-(b\eta_{1}+p_{e})^{2}]^{3}}>0$, and $\eta_{1}=1-\eta$. Thus, $\frac{\partial\pi_{r}^{N*}}{\partial\eta}<0$.

**Proof of Theorem 2**

According to $\frac{\partial U_{r}^{B}}{\partial p}=a-2p+w+bx-t(a-p+bx)$ and$\frac{\partial^{2}U_{r}^{N}}{\partial p^{2}}=-2+t<0$, we know that $U_{r}^{B}$ is a concave function about $p$. Let $\frac{\partial U_{r}^{B}}{\partial p}=0$; then, we can obtain the reaction function $p^{B}(x,w)=-\frac{a+w-at-bx-btx}{t-2}$. By substituting $p^{B}(x,w)$ into $\pi_{m}^{B}$, we are able to obtain the Hessian matrix $H(w,x)=\left[\begin{matrix} 2/(t-2) & -(b-p_{e})/(t-2) \\ -(b-p_{e})/(t-2) & -(t+2bp_{e}-2)/(t-2) \end{matrix}\right]$. Given$\frac{\partial^{2}\pi_{m}^{B}}{\partial w^{2}}<0$ and when $2(2-t)>(p_{e}+b)^{2}$, . Thus, $\pi_{m}^{B}$ is jointly concave in $w$ and $x$. According to $\frac{\partial\pi_{m}^{B}}{\partial w}=0$ and $\frac{\partial\pi_{m}^{B}}{\partial x}=0$, we have $w^{B*}=\frac{h(a+ep_{e}+c)(2-t)-[p_{e}(a+be)+bc](b+p_{e})}{2h(2-t)-(b+p_{e})^{2}}$, and$x^{B*}=\frac{(b+p_{e})(a-c-ep_{e})}{2h(2-t)-(b+p_{e})^{2}}$. Furthermore, we can obtain $p^{B*}=\frac{h[(3-2t)a+c+ep_{e}]-[p_{e}(a+be)+bc](b+p_{e})}{2h(2-t)-(b+p_{e})^{2}}$. Finally, we can obtain $q^{B*}$, $\pi_{m}^{B*}$, $\pi_{r}^{B*}$, and $U_{r}^{B*}$.

**Proof of Proposition 3**

$x^{B*}-x^{N*}=\frac{B_{2}(A-c)(hT-B_{1}^{2})-AB_{1}(hT-B_{2}^{2})}{(hT-B_{2}^{2})(hT-B_{1}^{2})}$. The sign of $x^{B*}-x^{N*}$ is determined by the sign of$B_{2}(A-c)(hT-B_{1}^{2})-AB_{1}(hT-B_{2}^{2})$. By solving $B_{2}(A-c)(hT-B_{1}^{2})-AB_{1}(hT-B_{2}^{2})>0$, we can obtain $c<A[1-\frac{B_{1}(hT-B_{2}^{2})}{B_{2}(hT-B_{1}^{2})}]$.

**Proof of Proposition 4**

The proof of Proposition 4 is similar to that of Proposition 3. Therefore, we omit it here.

**Proof of Proposition 5**

(1) $\frac{\partial\pi_{m}^{BS*}}{\partial\lambda}=\frac{h(a-c-ep_{e})^{2}(b+p_{e})^{2}}{2[2h(1-\lambda)(2-t)-(b+p_{e})^{2}]^{2}}>0$;

(2) $\frac{\partial\pi_{r}^{BS*}}{\partial\lambda}=\frac{h(a-c-ep_{e})^{2}(b+p_{e})^{2}[(b+p_{e})^{2}-2ht-h\lambda(8-6t)]}{2[2h(1-\lambda)(2-t)-(b+p_{e})^{2}]^{3}}$. The sign of $\frac{\partial\pi_{r}^{BS*}}{\partial\lambda}$ is determined by the sign of $(b+p_{e})^{2}-2ht-h\lambda(8-6t)$. Thus, if $\lambda<\frac{(b+p_{e})^{2}-2ht}{h(8-6t)}$, then $\frac{\partial\pi_{r}^{BS*}}{\partial\lambda}>0$; if $\lambda>\frac{(b+p_{e})^{2}-2ht}{h(8-6t)}$, then $\frac{\partial\pi_{r}^{BS*}}{\partial\lambda}<0$. In addition, given that $2h(1-\lambda)(2-t)>(b+p_{e})^{2}$, $(b+p_{e})^{2}-2ht<4h-4h\lambda-4ht+2h\lambda t$. Thus, $\frac{(b+p_{e})^{2}-2ht}{h(8-6t)}<\frac{h(4-4\lambda-4t+2\lambda t)}{h(8-6t)}$. According to $h[(8-6t)-(4-4\lambda-4t+2\lambda t)]>0$, we have $0<\frac{(b+p_{e})^{2}-2ht}{h(8-6t)}<1$.

**Proof of Theorem 3**

Solving the first and second order partial derivatives of $U_{r}^{BS}$with respect to $p$, we have $\frac{\partial U_{r}^{BS}}{\partial p}=a-2p+w+bx+c\lambda-\frac{t(2a-2p+2bx)}{2}$. Letting $\frac{\partial U_{r}^{BS}}{\partial p}=0$, We obtain the reaction function $p^{BS}(w,x)=-\frac{a+w-t(a+bx)+bx+c\lambda}{t-2}$. Substituting $p^{BS}(w,x)$ into $\pi_{m}^{BS}$, we can obtain the hessian matrix $H(w,x)=\frac{1}{2-t}\left[\begin{matrix} -2 & (b-p_{e}) \\ (b-p_{e}) & (2bp_{e}-h(1-\lambda)(2-t)) \end{matrix}\right]$. When $2h(1-\lambda)(2-t)>(b+p_{e})^{2}$, then $\left|\frac{\partial^{2}\pi_{m}^{BS}}{\partial w^{2}}\right|<0$, and . By solving $\frac{\partial\pi_{m}^{BS}}{\partial w}=0$ and $\frac{\partial\pi_{m}^{BS}}{\partial x}=0$, we can obtain $w^{BS*}=\frac{\begin{matrix} h(a+ep_{e}+c)(1-\lambda)(2-t) \\ -[p_{e}(a+be)+bc](b+p_{e}) \end{matrix}}{2h(1-\lambda)(2-t)-(b+p_{e})^{2}}-c\lambda$, and$x^{BS*}=\frac{(b+p_{e})(a-c-ep_{e})}{2h(1-\lambda)(2-t)-(b+p_{e})^{2}}$. Substituting $w^{BS*}$ and $x^{BS*}$ into $p(w,x)$, we have $p^{BS*}=\frac{\begin{matrix} h[(3-2t)a+c+ep_{e}](1-\lambda) \\ -[p_{e}(a+be)+bc](b+p_{e}) \end{matrix}}{2h(1-\lambda)(2-t)-(b+p_{e})^{2}}$. According to the equilibrium solutions, we can get the optimal market demand and the profit of the manufacturer and the retailer.

**Proof of Proposition 6**

(1) $x^{N*}-x^{BS*}=\frac{[(1-\lambda)hT-B_{2}^{2}]AB_{1}-(hT-B_{2}^{2})(AB_{2}-B_{2}c)}{(hT-B_{1}^{2})[(1-\lambda)hT-B_{2}^{2}]}$. The sign of $x^{N*}-x^{BS*}$ is determined by the sign of $[(1-\lambda)hT-B_{2}^{2}]AB_{1}-(hT-B_{1}^{2})(AB_{2}-B_{2}c)$. By solving $[(1-\lambda)hT-B_{2}^{2}]AB_{1}-(hT-B_{1}^{2})(AB_{2}-B_{2}c)=0$, we have $c_{4}=A[1-\frac{B_{1}[(1-\lambda)hT-B_{2}^{2}]}{B_{2}(hT-B_{1}^{2})}]$. Here, $A=a-ep_{e}$, $B_{1}=p_{e}+b\eta_{1}<B_{2}=p_{e}+b$, $T=2(2-t)$, $0<\lambda<1$.

(2) $\pi_{m}^{BS*}-\pi_{m}^{N*}=\frac{1}{2}\frac{(A-c)^{2}\lambda_{1}(hT-B_{1}^{2})-A^{2}(\lambda_{1}hT-B_{2}^{2})}{(\lambda_{1}hT-B_{2}^{2})(hT-B_{1}^{2})}$. The sign of $\pi_{m}^{BS*}-\pi_{m}^{N*}$ is determined by the sign of the numerator in the formula. By solving $(A-c)^{2}\lambda_{1}(hT-B_{1}^{2})-A^{2}(\lambda_{1}hT-B_{2}^{2})>0$, we can obtain $c<A[1-\sqrt{(\lambda_{1}hT-B_{2}^{2})/[\lambda_{1}(hT-B_{1}^{2})]}=c_{5}$, where $A=a-ep_{e}$, $B_{1}=p_{e}+b\eta_{1}$, $B_{2}=b+p_{e}$, $T=2(2-t)$, $\lambda_{1}=1-\lambda$.

(3)$\pi_{r}^{BS*}-\pi_{r}^{N*}=\frac{h(A-c)^{2}[2h(1-t)\lambda_{1}^{2}-(1-\lambda_{1})B_{2}^{2}](hT-B_{1}^{2})^{2}-2A^{2}h^{2}(1-t)(hT\lambda_{1}-B_{2}^{2})^{2}}{2(hT\lambda_{1}-B_{2}^{2})^{2}(hT-B_{1}^{2})^{2}}$. The sign of $\pi_{r}^{BS*}-\pi_{r}^{N*}$ is determined by the sign of the numerator in the formula. By solving $h(A-c)^{2}[2h(1-t)\lambda_{1}^{2}-(1-\lambda_{1})B_{2}^{2}](hT-B_{1}^{2})^{2}-2A^{2}h^{2}(1-t)(hT\lambda_{1}-B_{2}^{2})^{2}>0$, we have $c<A[1-\frac{\lambda_{1}hT-B_{2}^{2}}{hT-B_{1}^{2}}\sqrt{\frac{h(T-2)}{h(T-2)\lambda_{1}^{2}-(1-\lambda_{1})B_{1}^{2}}}]=c_{6}$. Here $A=a-ep_{e}$, $B_{1}=p_{e}+b\eta_{1}$, $B_{2}=b+p_{e}$, $T=2(2-t)$, $\lambda_{1}=1-\lambda$.

(4) According to (2) and (3), we can obtain $\pi_{m}^{BS*}>\pi_{m}^{N*}$ and $\pi_{r}^{BS*}>\pi_{r}^{N*}$ when $0<c<\min\{c_{5},c_{6}\}$.

**Proof of Corollary 1**

We substitute $x^{BS*}$, $w^{BS*}$ , $p^{BS*}$ and $x^{BS*}$ into $U_{r}^{BS}$. By solving $\frac{\partial U_{r}^{BS}}{\partial\lambda}=\frac{h(b+p_{e})^{2}(a-c-ep_{e})^{2}[(b+p_{e})^{2}-4h\lambda(2-t)]}{2[2h(1-\lambda)(2-t)-(b+p_{e})^{2}]^{3}}=0$, we have $\lambda^{R*}=\frac{(b+p_{e})^{2}}{4h(2-t)}$.

**Proof of Corollary 2**

Let $\lambda_{1}=1-\lambda$, $F=(a-c-ep_{e})^{2}$ ,$G=Ep_{e}$, $H=(b+p_{e})^{2}$, $I=2-t$. On the basis of Theorem 3, we substitute $\pi_{m}^{BS*}$ and $U_{r}^{BS*}$ into $\max_{\lambda} \Pi^{NB}(\lambda)=\pi_{m}^{BS*}*U_{r}^{BS*}$. Then, by solving $\frac{\partial\Pi^{NB}}{\partial\lambda_{1}}=0$, we can obtain $\frac{\partial^{2}f(\lambda_{1})}{\partial\lambda_{1}^{2}}=-2(16GI^{2}+5FI)h^{2}<0$, where $f(\lambda_{1})=-(16GI^{2}+5FI)h^{2}\lambda_{1}^{2}+2(8GI^{2}h^{2}+2GHIh+2FIh^{2}-FHh)\lambda_{1}+2GH^{2}+FHh-8GHIh=0$. Thus, if $f(\lambda_{1})=0$, then $\frac{\partial\Pi^{NB}}{\partial\lambda_{1}}=0$. According to $\frac{\partial^{2}f(\lambda_{1})}{\partial\lambda_{1}^{2}}=-2(16GI^{2}+5FI)h^{2}<0$, we know that $\frac{\partial f(\lambda_{1})}{\partial\lambda_{1}}=-2h^{2}(16GI^{2}+5FI)\lambda_{1}+2(8GI^{2}h^{2}+2GHIh+2FIh^{2}-FHh)$ is a decreasing function of $\lambda_{1}$ on $[0,1]$. $\left.\frac{\partial f(\lambda_{1})}{\partial\lambda_{1}}\right|_{\lambda_{1}=0}=2(8GI^{2}h^{2}+2GHIh+2FIh^{2}-FHh)>0$ and $\left.\frac{\partial f(\lambda_{1})}{\partial\lambda_{1}}\right|_{\lambda_{1}=1}=-2[8GI^{2}h^{2}+3FIh^{2}-2GHIh+FHh]<0$; thus, a unique $\lambda_{11}\in(0,1)$ that satisfies $\left.\frac{\partial f(\lambda_{1})}{\partial\lambda_{1}}\right|_{\lambda_{1}=\lambda_{11}}=0$, where $\lambda_{11}=\frac{8GI^{2}h^{2}+2GHIh+2FIh^{2}-FHh}{h^{2}(16GI^{2}+5FI)}$, exists. Therefore, if $\lambda_{1}\in[0,\lambda_{11}]$, then $f(\lambda_{1})$ is an increasing function of $\lambda_{1}$; if $\lambda_{1}\in[\lambda_{11},1]$, then $f(\lambda_{1})$ is a decreasing function of $\lambda_{1}$. According to $f(0)=2GH^{2}+FHh-8GHIh>0$, $f(1)=-FIh^{2}-FHh-2GH(2Ih-H)<0$, we know that a unique $\lambda_{1}^{*}\in(0,1)$ that can make $f(\lambda_{1})=0$ exists. By solving $f(\lambda_{1})=0$, we can obtain $\lambda_{1}^{*}=\frac{[2GIh(4Ih+H)+Fh(2Ih-H)]+\sqrt{[2GIh(4Ih+H)+Fh(2Ih-H)]^{2}+h^{2}(16GI^{2}+5FI)(2GH^{2}+FHh-8GIHh)}}{(16GI+5F)Ih^{2}}$. Thus, $\lambda^{NB*}=1-\lambda_{1}^{*}$.
